# Fluorescence polarization assay to detect the presence of traces of ciprofloxacin

**DOI:** 10.1038/s41598-020-61395-3

**Published:** 2020-03-12

**Authors:** Hiyam El Kojok, Nada El Darra, Mahmoud Khalil, Alessandro Capo, Angela Pennacchio, Maria Staiano, Alessandra Camarca, Sabato D’Auria, Antonio Varriale

**Affiliations:** 1 Department of Biological Sciences, Faculty of Science, Beirut Arab University, Beirut, Lebanon; 2 Beirut Arab University, Faculty of Heath Sciences, Tarik El Jedidah, Beirut, P.O.Box: 115020 Riad EL Solh 1107 2809, Beirut, Lebanon; 3 Institute of Food Science, CNR, Via Roma, 64, 83100 Avellino, Italy

**Keywords:** Biochemistry, Biotechnology, Fluorescence spectroscopy, Biochemical assays

## Abstract

Detection of ciprofloxacin residues in milk by sensitive and rapid methods is of great interest due to its use in the treatment of dairy livestock health. Current analytical approaches to antibiotics detection, are laboratory-based methods and they are time-consuming and require trained personnel. To cope this problem, we propose an assay, based on fluorescence polarization principle, able to detect the presence of ciprofloxacin in diluted milk sample without any pre-treatment. The proposed method is based on the use of ciprofloxacin-protein conjugate labeled with near infrared fluorescence dye, which upon binding to specific antibody causes an increase of the fluorescence polarization emission signal. The developed assay allows for the detection of ciprofloxacin at a concentration of 1ppb, which represents an amount lower than the maximum residual limit (MRL) of ciprofloxacin in milk, as set by the European Union regulation (100 ppb).

## Introduction

The antibiotics treatment plays a crucial role in dairy livestock health^1^. It is being used for therapeutic purposes as well as for improving breeding efficiency. However, this treatment can leave residues of antibiotics in foods of animal origin (milk and meat), and consequently an increase of the human exposure to antibiotics^2^. While assessing the usage of antibiotic residues, the exceptional use of such drugs was legally considered^3^. Hence, the risk of the presence of antibiotic residues in milk and dairy products could increase in case that a required elimination period of them in food is not properly defined.

Antibiotic residues in milk and dairy products present detrimental consequences on the consumer health, causing disturbances in the intestinal flora as well as allergic reactions that could lead to anaphylaxis^4^. Moreover, antibiotic residues might increase microbial resistance, a major health risk^5^. Among anti-bacterial agents used for the management of infections in dairy livestock, ciprofloxacin, belonging to the fluoroquinolone class, is the common antibiotic used in case of pulmonary, urinary and digestive infections^6^. However, ciprofloxacin could cause hypersensitivity in humans^7^. Due to the importance of considering the presence of ciprofloxacin in milk, a residue surveillance of anti-microbial presence is crucial to support food-farming practices, upraising consequently the food safety level. It is to be noted that the maximum residual limit (MRL) for ciprofloxacin in milk has been fixed at 100 ppb (Council Regulation EEC/2377/90). In Lebanon, few studies have investigated the presence of antibiotic residues in milk^8–10^. A recent study showed that ciprofloxacin and oxytetracycline are mostly found antibiotics in milk in Lebanon (personal communication).

Currently, laboratory-based methods, such as liquid chromatography-mass spectrometry (LC-MS), high performance liquid chromatography (HPLC)^11–13^ and microbiological assays^14^ are the common used approaches for ciprofloxacin detection. These methods have different restrictions that make them challenging to apply outside the laboratory. Therefore, there is a growing need for quick, easy, and not expensive methods to detect and manage ciprofloxacin residues in food matrices^15–18^.

Fluorescence polarization assays represent a valid alternative to detect chemical contaminants in food and milk samples^19^. In this work, a fluorescence polarization assay for the detection of the presence of ciprofloxacin in milk was developed using *ad hoc* synthesized fluorescence ciprofloxacin-conjugate (GlnBP-CPFX), covalently labeled with a near-infrared (NIR) fluorescence dye, and commercial monoclonal anti-ciprofloxacin antibody. The principle of the assay is that, at stable temperature and viscosity, the fluorescence polarization emission value is related on the molecular size of the excited molecule^20^. Thus, a competitive immunoassay has been deployed to directly detect ciprofloxacin residues in commercial diluted milk solution sample. The obtained results show a high sensibility of the assay (1.0 ppb) respect the maximum residual limit (MRL) set by the European Union regulation (100 ppb). Finally, it is worth to note that this method can be transferred into a hand-held device capable to acquire fluorescence polarization changes when ciprofloxacin derivative and anti-ciprofloxacin antibodies interacts.

## Methods

### Reagents

All purchased materials were the highest quality available. Ciprofloxacin CPFX, 1-[3-(Dimethylamino)-propyl]-3-ethylcarbodiimide (EDC) and Sodium phosphate were purchased from Sigma-Aldrich (St. Louis, MO, USA), Potassium dihydrogen phosphate was purchased from Applicam, Germany. Mouse monoclonal anti-ciprofloxacin antibody was purchased from Abbexa (Cambridge, UK). Goat polyclonal anti-mouse IgG-HRP conjugate (secondary antibody) was purchased from Abcam (Cambridge, UK). ECL detection reagents and Immobilon-P^SQ^ PVDF Membrane were purchased from Amersham Biosciences (GE Healthcare Switzerland) and from Merck, USA respectively. The Enzyme substrate 3,5-tetramethylbenzidine (TMB) was purchased from Sigma Aldrich while the Microplates (96-well), Nunc LockWell C8 MaxiSorp was purchased from Thermo Scientific. The fluorescent Amine-Reactive Dye CF647, Succinimidyl-ester was obtained from Biotium (Freemont, USA).

### GlnBP-CPFX conjugate preparation

The GlnBP-CPFX molecules was prepared by conjugation the CPFX to a recombinant glutamine-binding protein (GlnBP) isolated from *Escherichia coli*^21^. Briefly, the conjugate was synthesized through carbodiimide method described by^15^ with slight modifications. Ciprofloxacin 1.0 ml (26.2 mg/ml), was mixed with 1.0 ml of GlnBP (4.5 mg/ml) and EDC (314 mg/ml in 0.01 M Phosphate buffer, pH 5.0). The obtained mix reaction was allowed to occur at 28 °C for 2 h. Dialysis of the reaction mixture was carried out for two days in 0.01 M phosphate buffer, pH 5.0, and the dialyzing buffer was changed twice per day.

### Western blotting

Western blotting experiment was performed according to Varriale^19^. In brief, GlnBP, CPFX-GlnBP and CPFX antibodies (4 µg each) were separated by 12% SDS-PAGE and then transferred overnight at 4 °C onto a Polyvinylidene difluoride (PVDF) membrane. After this step, membrane was blocked using TBS containing 5% of milk for 30 min at room temperature, then washed with TBST (three washes for 10 minutes each time.) and incubated with purified mouse monoclonal CPFX antibodies (1:1000) for 1 hr. at 37 °C. After this incubation step, the membrane was washed (three times) and then incubated with goat anti-mouse IgG-HRP conjugate (1:5000) for 1 h at 37 °C. Finally the protein bands developed using ECL.

### Antibody titer determination

To determine the antibody titer according to de Champdoré^19^ an ELISA test was performed as described by Varriale^18^ with slight modifications. Each well of a 96-well plate was coated by CPFX-GlnBP antigen (0.00625 mg/ml) in bicarbonate buffer (0.05 mol/L, pH 9.6), with different dilutions (1/200, 1/400, 1/800 and 1/1600) at 4 °C overnight. As control, some wells were coated by coating buffer and other wells were coated by GlnBP protein. The wells were washed three times with washing buffer (PBS 0.01 mol/L pH 7.4 containing 0.05% Tween-20; PBST) and after the incubation with 200 μl/well of blocking buffer (0.02 mol/L PBS at pH 7.4 including 2% (w/w) glycine), at 37 °C for 2 h, the plate was rinsed three times. After this step, 100 μl of monoclonal antibodies of different dilutions were incubated in the coated wells at 37 °C for 1 h. The plate was rinsed (three times), goat anti-mouse IgG-HRP antibody (1:5000, 100 μl/well) was added, and the wells were incubated for 1 h at 37 °C. Finally, the enzyme substrate solution (TMB) was added (50 μl /well), and the wells were incubated at 37 °C, then color development was quenched by adding stopping solution H_2_SO_4_ (2 mol/L, 50 μl /well) after 10 min. A micro-plate reader was used to measure the absorbance at 450 nm.

### Fluorescence labelling of CPFX-GlnBP

The conjugate CPFX-GlnBP was labeled according to Varriale^18^. 100 μl of CPFX-GlnBP (5 mg/ml) in 1 mol/L sodium bicarbonate at pH 8.3 was mixed with the dye CF647 (molar ratio 1:12) and the reaction mixture was kept at room temperature for 1 hour. Separation of CPFX-GlnBP-CF647 from un-reacted probe was done by gel filtration (G25 Sepharose) and extensive dialysis against PBS 0.01 mol/L pH 7.4.

### Steady-state fluorescence measurements

Steady-state measurements were performed using a fluorescence spectrometer FP-8600 (Jasco-Japan). The polarization emission spectra of CPFX-GlnBP-CF64 were acquired setting excitation value at 650 nm and recording between 654 nm and 800 nm. In antibody binding experiments, the fluorescence spectra were carried out through incubation, overnight at 37 °C, of CPFX-GlnBP-CF647 with increasing concentrations of anti-CPFX. The antibody concentration tested was from 20 pM to 2000 pM in 0.01 mol/L PBS buffer at pH 7.4. The polarization fluorescence measurements were obtained with polarized filter, in excitation and emission, set at vertical position.

### Ciprofloxacin competitive immunoassay

A competitive polarization immunoassay with fixed concentration of antibody (1000 pM) and increasing concentration of un-labeled ciprofloxacin from 1 ppb to 150 ppb was performed. The fluorescence polarization measurements were performed in both PBS buffer (0.01 mol/L, pH 7.4) and milk diluted 100 times in PBS. The measurements were performed at the end of an overnight incubation at 37 °C with the unlabeled ciprofloxacin, and the excitation and emission polarized filters were set at vertical position.

## Results

**Figure 1:**
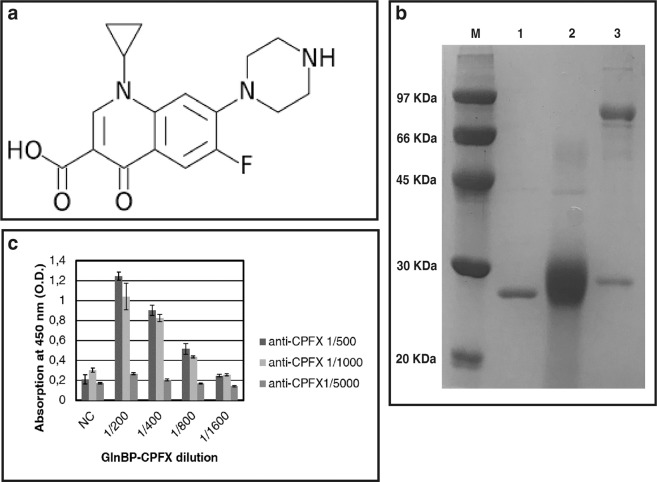
Chemical structure of ciprofloxacin (**a**) SDS-PAGE of the GlnBP, GlnBP-CPFX conjugate and monoclonal anti-CPFX (**b**) anti-CPFX titer (**c**).

In this study, we describe a fluorescence polarization assay for the detection of the ciprofloxacin. Ciprofloxacin is a fluoroquinolones (FQ) used in the management of pulmonary, urinary and digestive infections in animal livestock. As all antibiotic molecules, ciprofloxacin is a very small molecule (Fig. 1a) unable to elicit an immunological response in an organism. For the assay development, we selected specific commercial monoclonal antibody against the CPFX. In order to characterize the antibody binding features and avoid any cross-reactivity effects, we conjugated the CPFX to a protein carrier. For this purpose, according to Varriale^18^, the CPFX was conjugate to the recombinant GlnBP isolated from *E. coli* by the well-known conjugation processes using EDC^22,23^. At the end of the conjugation process, SDS-PAGE analysis of the GlnBP and GlnBP-CPFX conjugate was performed to verify the purity of the sample preparations and to evaluate the conjugation effect on the molecular weight of GlnBP (Fig. 1b). The obtained conjugate molecule GlnBP-CPFX, was labeled with a specific fluorescence probe (CF647) and the achieved fluorescence molecule (GlnBP-CPFX-CF647) was used for the development of the competitive fluorescence polarization immunoassay.

### Western blotting and ELISA results

To confirm the specificity of monoclonal antibodies versus the produced GlnBP-CPFX conjugate, western blotting and indirect ELISA tests were performed. In the western blotting experiments, the response was observed against GlnBP-CPFX, while a negative response was showed for GlnBP and BSA (data not shown). On the contrary, an ELISA test was done to evaluate the antibody titer. The value was calculate according to Di Giovanni^24^ and as presented in Fig. 1c, it was possible to perform the ELISA test with monoclonal anti-CPFX up to 1 to 1000 dilution.

### Competitive ELISA test

**Figure 2:**
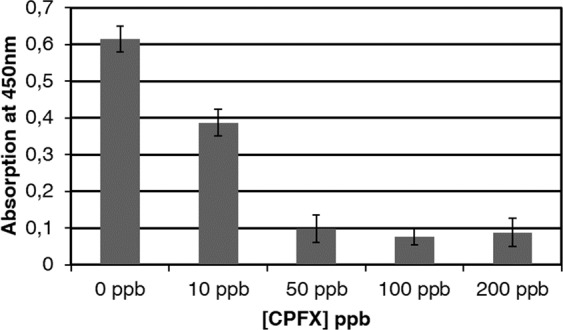
Competitive indirect ELISA test. The plate was coted with 0.00625 mg/ml GlnBP-CPFX and the anti-CPFX was diluted 1:1000. All the measurements were done in triplicate.

A competitive indirect ELISA was performed to set up a sensitive assay for CPFX detection. The plate was coated with fixed amount of GlnBP-CPFX (6,25 μg/μl) and incubated with anti-CPFX in presence of increased concentration of un-labeled CPFX. In Fig. 2 is reported the variation of the absorption at 450 nm as consequence of the increased concentration of the CPFX. The obtained data show a significant variation of the signal (about 1,5 times) a very low concentration of CPFX (10 ppb).

### Fluorescence polarization assay

**Figure 3:**
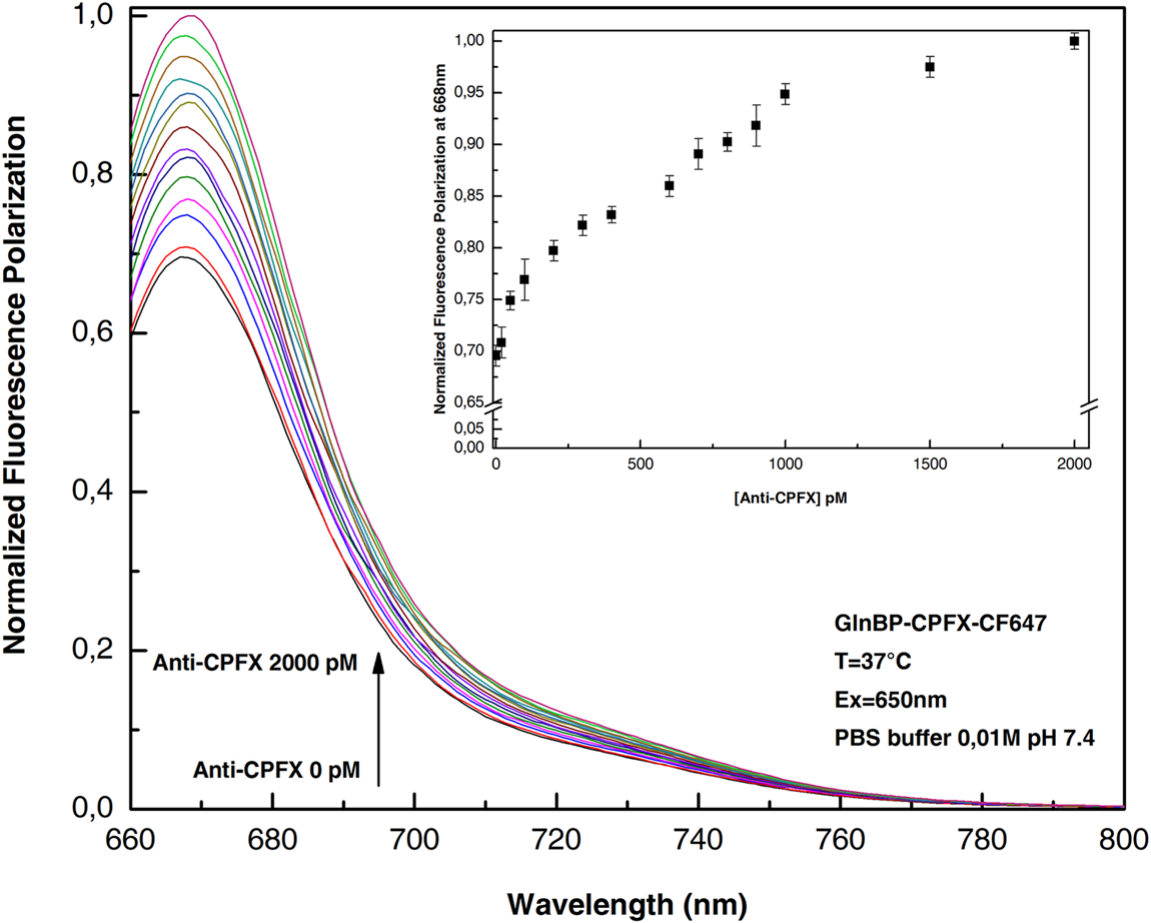
Fluorescence polarization emission spectra of ciprofloxacin-GlnBP-CF647 in the absence and presence of an increasing concentration of anti-ciprofloxacin. Variation of the maximum fluorescence value at 668 nm as a function of the antibody concentration (inset). All the measurements were done in PBS buffer 0.01 M pH 7.4 at 37 °C.

In Fig. 3 are depicted the polarized emission spectra of GlnBP-CPXF-CF647 acquired at 37 °C. The sample was excited at 650 nm and shows a maximum of emission centered at 668 nm. The anti-CPFX was added at concentrations from 20 pM to 2000 pM and the obtained results show an increase of the polarization signal as consequence of the increase amount of antibody in solution. In the inset of Fig. 3, is reported the variation of the maximum of polarized fluorescence emission at 668 nm versus the antibody concentration. The increase of intensity is registered and correlated to the addition of increased concentrations of antibodies.

### Competitive assay

**Figure 4:**
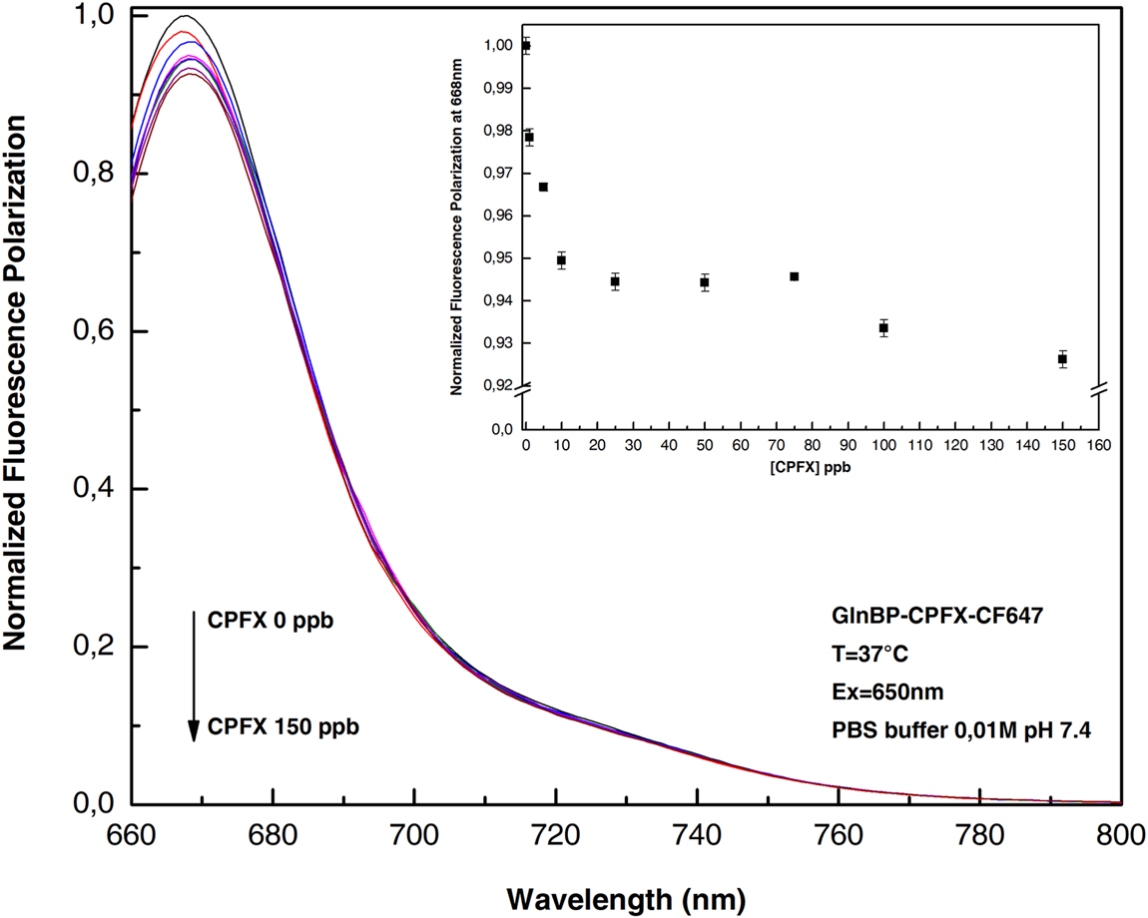
Fluorescence polarization emission spectra of ciprofloxacin-GlnBP-CF647 with increasing concentration of unlabeled Ciprofloxacin in the presence of 1000 pM anti-ciprofloxacin antibody. All the measurements were done in PBS buffer 0.01 M pH 7.4 at 37 °C.

A competitive fluorescence polarization immune-assay was performed to study the competition between the un-labeled CPFX and GlnBP-CPFX-CF647. For this purpose, samples of anti-CPFX antibody (1000 pM) were incubated in the presence of different amounts of un-labeled CPXF. The data reported in Fig. 4, show the reduction of polarized fluorescence emission at the increasing concentration of un-labelled CPFX. This effect, due by the competition between the anti-CPFX to both GlnBP-CPFX-CF647 and unlabeled CPFX present in solution, allows to detect traces of the CPFX in solution. Figure 4 (inset), reports the change of the fluorescence intensity as a function of CPFX concentration. The presence of a very low concentration of CPFX in solution produces a reduction of the fluorescence emission.

**Figure 5:**
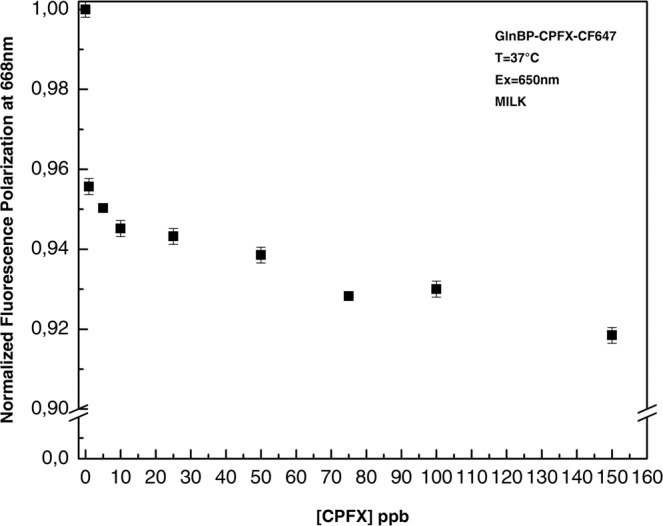
Dose-response curve of CPXF detection. Titration of fluorescence polarization immune assay with increasing concentrations of un-labeled CPXF. All the measurements were performed in milk diluted 100 times in PBS buffer 0.01 M pH 7.4 at 37 °C.

Finally, the developed assay was tested in real food matrices. A competitive experiment was performed diluting increased quantities of CPFX in diluted milk samples. Figure 5 shows the variation of the polarization fluorescence intensity as function of CPFX concentration in diluted milk (1:100). The results show that it is possible to detect less than 1.0 ppb of CPFX directly in the milk solution. This value is lower than the value reported in the EU regulation (100 ppb).

## Conclusion

Veterinary drugs residues, such as antibiotics, released in animal origin foodstuff have an important consequence for human health and so their detection has become the main aim of food safety control. In this work, we have described the development of a near-infrared fluorescence polarization assay for the detection of the antibiotic CPFX directly in diluted milk solution. The amount of antibiotics in food is rigorously regulated by the European Union and MRLs has been established. This value for ciprofloxacin in milk is 100 ppb (Council Regulation EEC/2377/90).

## Supplementary information

Supplementary information.
