# Supplementary material for: Fluorescence polarization assay to detect the presence of traces of ciprofloxacin
Source: Sci Rep. 2020 Mar 12;10:4550. doi: 10.1038/s41598-020-61395-3 (PMC7067807; doi:10.1038/s41598-020-61395-3)
Supplement: Supplementary file 1 — Supplementary information. [file 41598_2020_61395_MOESM1_ESM.docx]

Fluorescence polarization assay to detect the presence of traces of ciprofloxacin

Hiyam El Kojok^1^, Nada El Darra^2^, Mahmoud Khalil^1^, Alessandro Capo^3^, Angela Pennacchio^3^, Maria Staiano^3^, Alessandra Camarca^3^, Sabato D’Auria^3*^ and Antonio Varriale**^3^**

M A B C


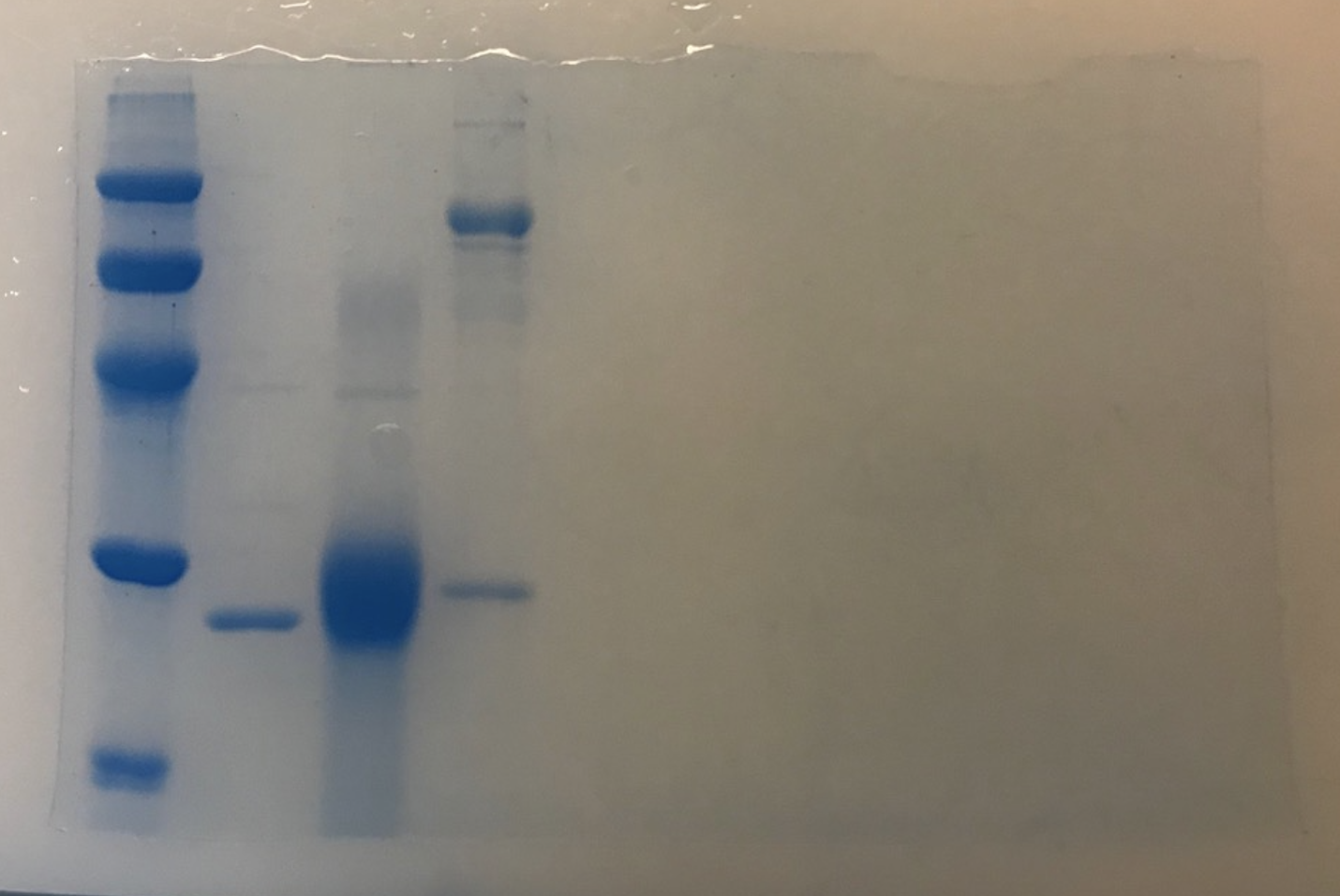


**FigureS1.** SDS-PAGE GlnBP (A), GlnBP-CPFX (B) and anti-CPFX (C) was purchased from Abexxa (UK). The antibody catherization was performed from the company Abbexa.
<https://www.abbexa.com/index.php?route=product/document/datasheet&product_id=16794>
